# Neuroimaging Evidence for Processes Underlying Repetition of Ignored Stimuli

**DOI:** 10.1371/journal.pone.0036089

**Published:** 2012-05-01

**Authors:** Eva Bauer, Helge Gebhardt, Christoph Ruprecht, Bernd Gallhofer, Gebhard Sammer

**Affiliations:** Cognitive Neuro Science at the Centre for Psychiatry, Justus Liebig University, Giessen, Germany; University of Groningen, Netherlands

## Abstract

Prolonged response times are observed with targets having been presented as distractors immediately before, called negative priming effect. Among others, inhibitory and retrieval processes have been suggested underlying this behavioral effect. As those processes would involve different neural activation patterns, a functional magnetic resonance imaging (fMRI) study including 28 subjects was conducted. Two tasks were used to investigate stimulus repetition effects. One task focused on target location, the other on target identity. Both tasks are known to elicit the expected response time effects. However, there is less agreement about the relationship of those tasks with the explanatory accounts under consideration. Based on within-subject comparisons we found clear differences between the experimental repetition conditions and the neutral control condition on neural level for both tasks. Hemodynamic fronto-striatal activation patterns occurred for the location-based task favoring the selective inhibition account. Hippocampal activation found for the identity-based task suggests an assignment to the retrieval account; however, this task lacked a behavioral effect.

## Introduction

Selective attention helps us to achieve efficient goal directed behavior. It describes the ability to focus on goal relevant attributes of our internal or external environment. Processing of selected attributes in comparison to unselected is enhanced [1]. But not only the selected and attended information, also unattended information is processed and influences processing of subsequent stimuli [2]. This was observed in experiments comparing response times for experimental conditions in which an ignored distractor was subsequently repeated as target (so called DT conditions, *d*istractor becomes *t*arget) with conditions of no repetition (control condition). Those targets were associated with increased response times, indicating that the ignored, unattended distractor was indeed processed [2]. This phenomenon was called negative priming (NP), and was subject of many studies in experimental psychology conducted over the last 20 years [3]. To understand selective attention, it is essential to study the psychological determinants and neural mechanisms in situations where previously ignored information becomes relevant. Different characteristics of processing can be expected with and without (i.e. the first and second display are not related) repetition of stimuli. So called NP response time effects (defined as increased time taken to respond to a target, which was presented as distractor before, compared to control conditions with no repetition) give evidence for the existence of different processes underlying DT and control trials (C = trials without repetition). They could be observed in several tasks, with various stimuli, response modalities and number of trials [4], demonstrating the basic nature of NP. Mainly two conceptually different paradigms are used for the investigation of DT processing, identity-based tasks and location-based tasks. However, it remained still unclear whether DT is processed identically or not in both kinds of task.

Identity-based tasks require responses according to a target-intrinsic feature, e.g. naming the target stimulus or naming the color of the target stimulus. In a typical identity-based task, two stimuli are presented simultaneously. One of them is marked as target and it has to be indicated by the subject. The other stimulus (distractor) has to be ignored. In so called *ignored repetition trials*, the distractor of the current display (prime) becomes the target stimulus in the following display (probe) [5]. This results in prolonged response times compared to conditions without identity repetition (behavioral NP effect) [6]. In a location-based paradigm, subjects are asked to ignore the distractor and indicate the on-screen location of a pre-specified target with help of an appropriate response device (button array, joystick, etc.). In DT trials, the probe target appears at the location previously occupied by the prime distractor. DT trials result in increased response times, which are explained by additional distractor processing demands [7]–[8]. However, the nature of these control processes is under discussion [9]. Amongst others, two major explanatory accounts have been suggested in the context of NP research, the ‘selective inhibition’ account [10] and the ‘episodic retrieval’ account [11]–[12].

### Selective Inhibition Account

Houghton and Tipper [10] proposed the selective inhibition account, claiming that processing of DT situations involves cognitive inhibition. According to them, target selection in the prime display is based on persistent inhibition of the prime distractor. In other words, with the end of prime presentation inhibition decays gradually but does not resolve immediately. Inhibition is still active during the subsequent presentation of the probe. Accordingly, response selection is impaired and response times are increased. The involvement of inhibitory processes in the explanation of the NP phenomenon – at least for location-based tasks – is supported by several studies [5], [9], [13]–[15]. The subject of inhibition (e.g. response, perceptual pattern) is still discussed; for more information see section ‘Integrative Accounts’. But how exactly can we understand processes involved in DT situations according to the inhibitory approach? As inhibitory processes decay gradually and do not dissolve with offset of the prime, inhibition of the prime distractor is still active at the time of probe presentation. For this reason the probe target must be presented in a certain timeframe to reveal a NP effect [7]. And probe targets of DT trials in comparison to control probe targets are particularly ‘weak’, because their identity/location are similar or even identical to those of the *previously inhibited* prime distractor. Thus, in DT trials in comparison to control trials increased inhibitory processes again suppress the ‘predominant’ probe distractor [16]. Concluding, increased activation of brain areas associated with inhibition processing can be expected in DT probe trials. Naturally inhibition is not the only relevant process having an impact on performance. Whenever a prime distractor becomes a target conflict may arise. Thus, correlates of *conflict* have to be considered particularly in brain activation studies on DT situations.

Inhibitory processes correlate with distinct neural activation patterns. Cognitive inhibition is characterized by activation of a fronto-striatal network, consisting of frontal cortical structures, the striatum comprising putamen and caudate nuclei (NC), as well as the globus pallidus [17]. Especially the inferior frontal cortex plays an important role for cognitive inhibition, as it was for instance demonstrated by the investigation of switching tasks provoking Stroop-like interference [18]–[20]. In a single case lesion study, damage to the right frontal opercular part was in correlation with impaired performance in several attention tasks, which all required active inhibition of irrelevant signals [21]. However, active memory maintenance was not impaired in that patient.

The anterior cingulate cortex (ACC) has been found to correlate with levels of conflict [22]–[26]. Consequently, it can be argued, that DT probe trials most likely should be accompanied by increased activation of the ACC [1].

Concluding, in DT trials both fronto-striatal and medial frontal brain activation can be expected representing cognitive inhibition and conflict processing.

### Episodic Retrieval Account

Neill and Valdes [11] proposed an alternative account to explain the NP effect, the episodic retrieval account [11]–[12]. This account is based on the *Instance Theory of Automatization* by Logan [27]. Repeated task performance is associated with a change from rule-based algorithmic processing to episodic memory retrieval of previous occasions for a given stimulus and its representations. Episodic retrieval processes allocate a potential shortcut to prior solutions that potentially lead to automatization, i.e. subjects become faster but less variable in reacting [27]. This approach provides an alternative explanation of DT performance. The probe target was presented as a distractor within the prime display. Consequently, it is associated with a nonresponse (‘do not react’). The retrieved episode now is tagged with the wrong information (‘do not react’ instead of ‘react’). Supplemental retrieval efforts are necessary to search for an episode that is effectively linked to the probe target response. This supplemental retrieval processes or switching back to slower algorithmic processing results in increased response times for DT trials [28]. Just like the inhibition account, the episodic retrieval account has been subject of a broad debate. Various attempts have been made to improve the approach by integrating substantial findings. One of those variants is the ‘stimulus-response retrieval’ account by Rothermund, Wentura and De Houwer [29]. It is assumed that the prime *distractor* becomes associated with the prime *response* (e.g. ‘upper left button’) instead with nonresponse information (e.g. ‘do not react’). In contrast to Neill’s account, the stimulus-response retrieval account treats NP as a pure memory phenomenon and contains no elements of the selective attention account, for instance marking the distractor as irrelevant.

Egner and Hirsch [1] proposed that episodic retrieval might be attended by conflict processing. Retrieved probe target episodes are associated with ‘do not respond’. This tag is in conflict with the actually required response (‘react’). Just as for the selective inhibition account, a participation of the ACC in DT situations is quite expectable. However, for both accounts conflict is not absolutely mandatory. The episodic retrieval account particularly predicts involvement of retrieval processes on DT trials. Brain activation patterns associated with retrieval are often found in medial temporal regions, specifically the hippocampus [30]–[35]. Accordingly, both hippocampal and ACC activation are in consistency with the episodic retrieval account.

### Integrative Accounts

Principally, NP can engage on each processing step, i.e. perception, representation, and response. However, on which of the processing steps inhibition or retrieval become activated and what the preconditions are, is not understood yet [36]. Various results were discussed either in favor of the inhibition account or in favor of the episodic retrieval account [9], [36]. For instance, the finding of missing NP effects in absence of a probe distractor was suggested to giving evidence against the inhibition account. In 2011, Frings and Spence [37] reported about a NP effect in absence of a probe distractor when manipulating perceptual and conceptual processing difficulty. The authors see the results to be in agreement with both accounts. However, while the episodic retrieval account predicts the results of this study, the inhibition account does not provide clear predictions here [37].

Summarizing, none of the proposed accounts allows an exhaustive explanation of the data. Alternative accounts have been proposed, e.g. the feature mismatch theory to explain prolonged response times for DT situations in location-based tasks. According to the feature mismatch account, location-based NP effects result from the occupation of one and the same visual location by different stimuli on prime and probe. This causes feature mismatch in DT situations, wherefore they are processed less efficient compared to control trials. Although the episodic retrieval and the inhibition account have been considered as contradicting for many years, integrative accounts have been suggested. Among others, May, Kane and Hasher [4] proposed that NP effects are more likely to reflect inhibitory processes than retrieval processes under certain circumstances. Difficulty of target identification, ratio of target repetition, and response mode (yes-no decision vs. lexical decision) were mentioned to be notable factors. Tipper [9] expressed the idea that retrieval processes *as well as* inhibitory mechanisms play a central role in the processing of DT trials simultaneously. Episodic retrieval serves a backward processing mode, which is initiated by the occurrence of the probe stimulus. Inhibition is a forward acting process, beginning with target selection. A bi-directional process is proposed. Neill [36] speculated that NP of perceptual and conceptual representations is caused by episodic retrieval, and NP of responses is caused by inhibition.

To sum up, the processing mode involved in DT trials is still under discussion. Different neural networks have been associated with cognitive inhibition and episodic retrieval. Imaging brain function can contribute to identify the mechanisms underlying the processing of DT situations.

### Identity- and Location-based Tasks

Different outcome parameters can be used to detect differences between DT and C trials. In the ‘classical’ NP literature, response times indicate the NP effect. Electroencephalography (EEG) and fMRI was used to investigate the psychophysiological correlates of DT processing.

#### Behavioral studies

Both, location-based and identity-based DT conditions have been subject of psychological research since many years. Only a few attempts have been made to compare the two types of tasks directly. The results of comparative population studies gave evidence that identity-based and location-based DT situations are processed differently. According to May et al. [4], the NP effect cannot consistently be found in all populations for both paradigms, although both represent DT conditions. In subjects suffering from Alzheimer’s Disease [38]–[39] or Parkinson’s Disease [40]–[41], in children, and less constantly in older adults [42]–[43] disappearance of the NP effect in identity-based tasks but not in location-based tasks was reported. These findings suggest at least partly different underlying mechanisms for the two kinds of DT situations [4], [44]. However, no double dissociation was reported. The single dissociation observed most likely reflects varying degrees of task complexity [45]. Thus, the results of these comparative population studies do not imperatively imply different processing modes for identity- and location-based DT trials.

The strong generalizability reported for the NP effect implies common processing modes for both tasks. The NP effect can be generalized for various stimuli like pictures, words, letters, and Stroop color words. NP effects have also been reported for several different tasks like naming, making lexical decisions or classification, for various response modalities like spoken and manual responses, and for different total number of trials [4]. Even changes of response modality (e.g. key press to verbal naming) or of task type (e.g. from naming to categorization) between the prime and probe trial did not dissolve the NP effect [46].

#### EEG studies

Kathmann et al. [47] compared location- and identity-based tasks using event-related brain potentials. For the location-based DT condition enhanced P3 latency and reduced peak-to-peak amplitudes of the P1–N1 complex was associated with early inhibition of sensory processing and slowing of the stimulus evaluation process. For the identity-based DT condition larger P3 amplitudes were associated with increased attentional resources necessary for processing the probe targets. However, results of this study did not favor one of both accounts.

In the few published within-subject studies comparing location- and identity-based tasks, both tasks differed with regard to various experimental or task features. In a study examining event-related potentials Gibbons [3] tried to overcome those shortcomings. Gibbons used a paradigm allowing for direct comparison of identity- and location-based DT trials. He found differing brain potentials for the two types of DT trials. Enhanced N2 found for location-based DT gave evidence for the inhibitory account; enhanced N440 for the retrieval account. ERPs for the identity-based DT condition did not even differ from the control condition.

#### fMRI studies

To our knowledge, there are no publications directly comparing identity- and location-based tasks in a within-subject design using imaging techniques. The neural correlates of either identity-based DT trials or location-based DT trials were investigated only in a few studies.

Three imaging studies dealt with location-based paradigms. Wright et al. [48] found occipito-temporal and fronto-parietal activity for the DT (compared to C) condition. Fronto-parietal activity was shown particularly in the superior, inferior, and medial frontal gyri as well as in inferior parietal regions. This activity was assigned to inhibitory processing. Activation in the parietal association cortices as well as in the occipito-temporal cortices was interpreted as being ‘task-specific’. Krueger, Fischer, Heinecke, and Hagendorf [49] found activation in the dorsolateral prefrontal cortex (DLPFC) and in inferior parietal regions. These results were interpreted as being in line with the inhibitory account. DLPFC activation was assigned to top-down allocation of attentional resources, the parietal activation to mechanisms of selective attention. Vink et al. [15] found an increased BOLD signal in the putamen and in the supplementary motor area for location-based DT trials. Contrary to Krueger et al. [49] and Wright et al. [48] a decreased BOLD signal in the superior parietal lobe was reported. However, the divergent results can be explained by the use of a size discrimination task; for more discussion see [48].

The results of these location-based studies were suggested to be in accordance with the selective inhibition account by the authors. However, there is a lack of converging results, which in addition do not harmonize with the above-mentioned predictions for the selective inhibition account. Vink et al. [15] show evidence for striatal involvement, Wright et al. [48] for the right inferior frontal gyrus, and Krueger et al. [49] neither. Part of the problem is that Vink et al. [15] and Wright et al. [48] used a set of regions of interest (ROI) that may have prevented full identification of the putative frontal-striatal inhibitory network.

Two fMRI studies used an identity-based paradigm to study DT situations within Stroop-tasks. The study by Steel et al. [50] was seriously criticized for its design and lacking power [1], so we only refer the study by Egner and Hirsch [1]. These authors interpreted activation found in the DLPFC and the thalamus as consistent with the episodic retrieval account. DLPFC activation was enhanced for positively primed trials (target repetition). Since the right DLPFC is known to support monitoring and evaluation of information retrieved from episodic memory [51]–[52], this result was quite expectable because repetition processing nearly always involves retrieval. Thus, the DLPFC reflects cognitive control processes involved in retrieval rather than episodic retrieval processes per se. Increased activation in the ACC and the medial aspect of the superior frontal gyrus was at a lenient statistical threshold (p<0.01, uncorrected). However, activation of the superior frontal gyrus was suggested to be in association with increased conflict caused by the retrieved prime distractor.

To sum up, different activation patterns have been found in the cited imaging studies. Findings associated with location-based paradigms were interpreted within the meaning of the inhibitory account. Findings associated with identity-based paradigms were seen in accordance with the retrieval account. However, no conclusion can be drawn about the differences or similarities in processing of identity-based DT trials and location-based DT trials. None of the studies was designed for this purpose. No fMRI studies aiming a direct comparison using within-subject designs have been conducted. The experimental paradigms were significantly different; different ROI were used to search for brain activation impeding the post-hoc comparison of similarities in brain activation for the two tasks.

### Variants of NP

In addition to DT trials where only the prime distractor is repeated, we implemented also trials in which the prime target was repeated as probe distractor (condition DTTD; the prime *d*istractor becomes the probe *t*arget and the prime *t*arget becomes the probe *d*istractor). The reason for that was a report of Stadler and Hogan [53] who found stronger effects on behavioral level (prolonged response times) for the DTTD condition than for the DT condition. This implicates that DTTD probes are processed less effective than probes of both the C and the DT condition. Under the assumption that strong effects on behavioral level correlate with greater effects on the neural level, adding such a condition facilitates the detection of differences between ignored repetition trials and control trials on neural level [54]. According to the selective inhibition and the episodic retrieval account, quantitative changes rather than qualitative differences can be expected when comparing DTTD and DT situations. Regarding the episodic retrieval account, it is more likely that the probe stimuli trigger retrieval processes if two repeated stimuli are shown during the DTTD trial, compared to the DT situation where the probe consists of only one repeated stimulus. Regarding the inhibitory account, the additional change from the prime target to the probe distractor should certainly recruit supplementary inhibition [55]. Gibbons [3] reported slight differences between DT and DTTD conditions for ERPs, but not for response times. Matches and differences were found for DT and DTTD using location-based tasks. For identity-based tasks differences in processing of DT and DTTD trials were reported.

### Aims of the Present Study

Differences between processing of probe targets, which have been presented as distractor before (both conditions DT or DTTD) and probe targets, which have not been repeated (C), are indicated by increased response times. Which processes underlie processing of DT and DTTD trials is still under debate. Frequently discussed accounts are the inhibition and the episodic retrieval account. Furthermore, it remained unclear until now whether or not location-based and identity-based paradigms are mediated by different processes. Given these considerations, the present study aimed to investigate whether or not DT and control situations are processed similarly on neural level. Regarding the studies on behavioral level, differences between DT and C trials should be observable. The large body of recent NP literature has not proofed ability to clarify whether inhibition associated activation or episodic retrieval associated activation can be expected. Thus, we focus on fronto-striatal regions including ACC, being in accordance with the selective inhibition account. Equally, hippocampus and ACC are chosen as candidate regions with regard to the episodic retrieval account. The strong generalizability of the behavioral NP effect is most likely based on a common ‘mechanism’ holding for both tasks. Regarding the DTTD situations, we assume no general differences in the neural activation compared to DT situations (no qualitatively distinct processes). However, the effects are expected to be stronger for DTTD than for DT trials. Activation in similar brain regions are expected for DT and DTTD compared to C trials for each of the two paradigms (location-based and identity-based) used in this study.

Using an fMRI-adapted version of a design developed by Gibbons [3], task-specific activation is not expected, because both tasks were identical with respect to stimulus presentations. FMRI recordings were used to provide further evidence regarding the processing of distracting information in case of DT/DTTD situations. The study was done in healthy subjects in order to establish the paradigm for further research.

## Results

### Behavioral Data

As the error frequencies were negligible, only descriptive statistics are reported for the identity-based task (C: *M* = 0.82, *SD* = 0.94; DT: *M* = 0.68, *SD* = 0.82; DTTD: *M* = 0.82, *SD* = 1.25; TT: *M* = 0.36, *SD* = 0.87) and the location-based task (C: *M* = 0.80, *SD* = 0.94; DT: *M* = 0.68, *SD* = 0.82; DTTD: *M* = 0.5, *SD* = 0.84; TT: *M* = 0.36, *SD* = 0.78).

For the analysis of main effects in response times, a one-way ANOVA for repeated measures (including C, DT, DTTD, and TT) was conducted for the location-based task and the identity-based task separately. Subsequently, two-sided paired *t*-tests containing the four conditions were conducted for both tasks separately. The one-way ANOVA for repeated measures was significant for the location-based task (*F*(1,27) = 7.17; *p* = .0002). Post-hoc *t*-tests revealed significant differences between the conditions C and DT (*T*(27) = −2.89; *p* = .007), and between C and DTTD (*T* (27) = −4.08; *p* = .0003). Differences between the condition C and TT (*T* (27) =  0.95; *p* = .35), as well as DTTD and DT (*T* (27) = 1.43; *p* = .16) were not significant. For the identity-based task, the one-way ANOVA for repeated measures indicated significant differences between conditions (*F*(1,27) = 19.21; *p*<.0001). Post-hoc *t*-test showed significant differences between the conditions C and TT (*T* (27) = 5.62; *p*<.0001). No significant differences were detected between the condition C and DT (*T* (27) = 0.28; *p* = .78), C and DTTD (*T* (27) = −1.19; *p* = .24), as well as DT and DTTD (*T* (27) = 1.02; *p* = .31). However, response times in the DT condition were in 19 of 28 subjects longer than for the C condition (sign test: *Z* = 1.70; *p* = .089). See Table 1 for exact values.

**Table 1:** Averaged medians of response times in milliseconds for conditions C, DT, DTTD, and TT of the identity-based task and the location-based task with the according standard-deviation (SD).

	identity-based	location-based
C	755 (76)	532 (73)
DT	752 (83)	542 (73)
DTTD	763 (83)	548 (76)
TT	688 (102)	527 (63)

### Functional Imaging Data

We conducted a conjunction analysis to learn whether idDT and loDT are at least partially processed by an identical network. Surprisingly, we could not find significant activation in any ROI. Due to the identical stimulation in both tasks, it was for the first time possible to contrast the DT conditions between the two tasks and to test directly for differences in processing. We found no significant activation for the contrast (loDT – loC) – (idDT – idC), but a tendency in the right putamen (lo for *lo*cation-based, id for *id*entity-based). However, for the inverse contrast (idDT – idC) – (loDT – loC) we found a tendency for higher activation in the right hippocampus (see Table 2).

**Table 2:** Proposed localization and statistics of the peak voxels within the respective ROI for the contrasts ((idDT – idC) – (loDT – loC)) and ((loDT – loC) – (idDT – idC)).

contrast	brain structure	x	y	z	*Z*_max_	*p*_corr_
(idDT – idC) –(loDT – loC)	*R hippocampus*	*24*	*−22*	*−17*	*2.73*	.*096*
(loDT – loC) –(idDT – idC)	*R putamen*	*24*	*11*	*−8*	*2.73*	.*089*

*Note.* The threshold was *p_corr_*<.05 (FWE-corrected according to SPM8, small volume corrected). All coordinates (*x, y, z*) are given in MNI space. L = left, R = right. Marginal significant ROI are printed in italics.

**Figure 1:**
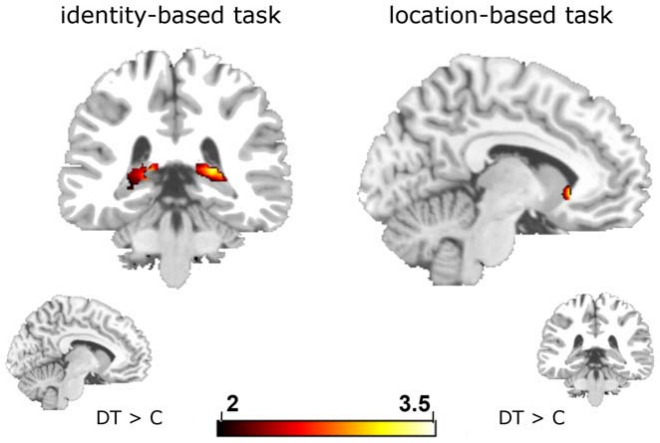
Neural activation for identity- and location-based priming. The contrast (DT – C) is presented, respectively. For coronal view the brain slice with y = −35 and for axial view with z = −7 is presented. For illustration reasons, data were thresholded at *T* ≥ 2.0.

To reveal specific activation of the two tasks, we performed separate analysis for the identity-based task and the location-based task. Computing the contrasts (idDT – idC) and (loDT – loC) we were able to study activation patterns for idDT and loDT, respectively. In ROI analyses for loDT we found activation in left NC and marginal significant activation in the left ACC (Table 3, Figure 1). Interestingly, the activation in the left ACC was positively correlated with the behavioral NP effect, but not the striatal activation.

**Table 3:** Localization and statistics of the peak voxels within the respective ROI activated during the identity- and location-based DT and DTTD trials.

contrast	brain structure	*x*	*y*	*z*	*Z*_max_	*p*_corr_
idDT – idC	R hippocampus	24	*−*40	7	3.31	.021
	L hippocampus	*−*18	*−*40	4	2.96	.054
	*R putamen*	*30*	*−19*	*1*	*2.96*	.*089*
	*R pallidum*	*30*	*−13*	*−5*	*2.37*	.*096*
	*R frontal inf. p.t.*	*39*	*38*	*1*	*3.21*	.*096*
idDTTD – idC	L pallidum	*−*27	*−*10	*−*5	3.01	.034
	L frontal inf. p.o.	*−*54	11	22	3.48	.041
	R frontal inf. p.t.	39	32	16	3.63	.053
	*L hippocampus*	*−27*	*−13*	*−11*	*3.14*	.*077*
	*L putamen*	*−30*	*−4*	*−5*	*3.09*	.*082*
	*L frontal inf. p.t.*	*−54*	*17*	*19*	*3.40*	.*089*
loDT – loC	L NC	*−*9	23	*−*5	3.06	.051
	*L ACC*	*−3*	*44*	*16*	*3.29*	.*064*
loDTTD – loC	R NC	21	26	7	3.64	.032
	L ACC	*−*6	32	*−*2	3.51	.045
	*L pallidum*	*−12*	*2*	*−5*	*2.62*	.*076*
	*R frontal inf. p.o.*	*48*	*17*	*31*	*3.31*	.*082*

*Note.* The threshold was *p_corr_*<.05 (FWE-corrected according to SPM8, small volume corrected). All coordinates (*x, y, z*) are given in MNI space. L = left, R = right, inf. p.t. = inferior pars triangularis, inf. p.o. = inferior pars opercularis. Marginal significant ROI are printed in italics.

IdDT showed activation patterns in the hippocampus; fronto-striatal activation patterns did not reach significance (Table 3, Figure 1). None of the activation patterns was correlated with the behavioral NP effect for the identity-based task. As we found activation in the hippocampus, pointing to retrieval processes taking place, we decided to conduct the contrast (idTT – idC) in addition. This way we were able to strengthen our assumption that hippocampal activation reflects retrieval mechanisms triggered by stimulus repetition. Indeed, we found activation in the right hippocampus (*x* = 24, *y* = −13, *z* = −23; *T* = 3.67; *p* = .025). We conducted several analyses to understand how DTTD situations are processed. For the identity-based task, the contrast (idDTTD – idC) revealed significant fronto-striatal activation (see Table 3). For the location-based task, the contrast (loDTTD – loC) was associated with neural activation in the NC and the ACC (see Table 3). To test for differences in the processing of DT and DTTD situations, we conducted the contrasts ((DT – C) – (DTTD – C)) and ((DTTD – C) – (DT – C)) separately for the two tasks. No significant differences were detected.

## Discussion

In the present study, we aimed to distinguish the neural correlates of DT trials in both a location-based and an identity-based task. Within-subject comparisons were conducted; activation associated with task presentation and response manipulation was eliminated by subtraction design. The first issue of interest was to clarify whether there exist brain structures equally serving both tasks. We conducted a conjunction analysis to answer this question. In a second step, a direct comparison of DT trials associated hemodynamic activation of both priming tasks was computed. In a third step we studied hemodynamic correlates of DT trials separately for each task. Furthermore, analyses of the DTTD trials were performed, and activation in DTTD trials was compared with activation in DT trials. Both types of tasks were studied using to some extent identical arrangements of stimuli (two numbers presented simultaneously on two of four possible locations). They differed in instructions, sequences, and composition of stimuli. Confounding effects by variations of the stimulus features and timing parameters have been avoided. Results particularly reflect effects of location or identity of the stimuli.

### Location-based Task

Applying a task adopted from Gibbons [3], we found significant differences in response times between the DT/DTTD condition and the C condition for the location-based task. This indicates that processing of DT/DTTD trials is altered in comparison to C trials. Brain imaging was used to investigate the underlying mechanisms of behavioral DT task effects.

#### DT trials

In analysis of the location-based DT trials we found activation in the NC which is part of fronto-striatal circuits. Activation in fronto-striatal networks has been reported in many studies investigating inhibitory processing [61]–[63]. The NC is a key structure of the fronto-striatal network mediating dopaminergic availability necessary for cognitive and especially executive functioning. Cools, Ivry and D’Esposito [64] showed that patients with striatal but no frontal lobe lesions were impaired in switching between concrete sensory stimuli indicating that the striatum plays a major role for flexible control functions associated with the selection of behaviorally relevant stimuli. The limited number of analyzed trials (maximum 18 per subject) and the rather large masks for the frontal ROI may have lead to lacking statistical power to detect frontal involvement. In location-based DT situations, inhibition of the predominant probe distractor may cause forced inhibitory mechanisms compared to the control condition. We found marginal significant activation of the ACC, and more interesting a significant correlation between the response behavior and AAC activation. The higher the behavioral NP effect was the stronger was the hemodynamic activation of the ACC. This association between the behavioral and neural activity clearly supports the inhibitory concept of DT processing. Higher conflict is related to more pronounced behavioral NP effects.

The lack of hippocampal involvement indicates that retrieval processes do not play a major role in location-based DT situations. As explicated in the Introduction paragraph, other studies examining location-based DT situations with fMRI concluded from their results an association with the inhibitory account. Gibbons [3] found enhanced selection negativity using EEG, which supports the view of persisting inhibition. Concurrently, he postulated evidence for brain potentials representing conflict at *later* stages of the probe, linking his data to the retrieval account.

However, the current study was not designed to differentiate between the feature mismatch account and other accounts. The inclusion of appropriate experimental conditions would have been necessary to study whether the feature mismatch of prime distractor and probe target *or* the inhibition of the prime distractor determines the NP effect.

#### DTTD trials

No significant differences between DT and DTTD conditions were found for the location-based task on behavioral and neural level. The neural activation patterns for the contrasts (loDT – loC) and (loDTTD – loC) were very similar. Results of this study indicate that inhibitory processes play a dominant role for both conditions. For DTTD situations significant activation in frontal areas was shown. However, only marginal significant activation was found there for DT situations. This strengthens the previously described view of processing of situations, in which distractors are presented as targets afterwards.

Gibbons et al. [54] found differences in EEG examining DTTD and DT situations. However, the method they applied is much more sensitive in the detection of small differences in neural processing, especially with respect to time resolution. Time resolution is much higher in EEG; spatial resolution is higher in fMRI. Results of EEG and fMRI studies are only comparable to a certain degree. The fMRI results of this study show that DT and DTTD are basically processed by the same brain structures. The general mechanisms acting in DT and DTTD situations as can be detected by fMRI are at least similar and involve inhibition. Other details of processing, as for instance timing of inhibitory processes and the stimuli which they act on, nonetheless might be different as Gibbons et al. suggest.

### Identity-based Task

For the identity-based task, no reliable behavioral NP effect was found. A reason therefore could be that the stimuli were presented on relatively distant locations. Behavioral NP effects for identity-based priming may depend on highly salient prime distractors, which may be best perceived when target and distractor are very close to each other or even overlapping [54]. Nevertheless, for direct comparisons it was necessary to design identical display pictures for both the location-based and the identity-based task. Distinct neural activation patterns were found when comparing the condition idDT/idDTTD with idC.

#### DT trials

Comparing the condition DT with the condition C for the identity-based task, increased activation in the hippocampal area was found as anticipated. This pattern was improved when contrasting the identity-based DT condition and the location-based DT condition directly. Hippocampal activation, which is associated with memory retrieval [30], [32]–[35] was marginally more pronounced in identity-based DT situations. To investigate whether hippocampal activation is repetition-sensitive, an additional analysis was conducted, namely the contrast (idTT – idC). In both conditions idTT and idDT, stimuli are repeated and initiate retrieval processes. Hippocampal activation for both, DT and TT trials of the identity-based task, would indicate that this activation is linked to repetition. Indeed, we found hippocampal activation for the identity-based TT condition. Other regions, which showed small activation for the identity-based DT condition (putamen, pallidum and inferior frontal pars triangularis of the right hemisphere), did not show up in the analysis of TT. In principle are the results of this study in accordance with Gibbons [3], who reported evidence for retrieval processes in identity-based DT trials using EEG.

It remains unclear why hippocampal activation was evident for DT compared to the control condition C in the identity-based task. Since no reliable NP effect on behavioral level was found for the identity-based task, the source of the brain activation cannot be stated without doubt. One explanation could be that hippocampal activation may particularly reflect supplemental retrieval efforts needed to search for an episode, which is consistent with the probe target, as the episodic retrieval account would predict. Here, this supplemental effort may not have been that high and had therefore no impact on response times in the DT condition. An alternative explanation is that the probe distractor in the identity-based DT condition did not serve as distractor, but rather as a cue, resulting in neural activation similar to the TT condition. The missing positive correlation between the hippocampal activation and the behavioral NP effect supports this argumentation. Further studies using an optimized identity-based task that is capable to produce a reliable behavioral NP effect are necessary to clarify this issue.

#### DTTD trials

DTTD trials were thought to improve insight in the processing of situations where distractors become targets later on. Unexpectedly, the DTTD condition, which has been associated with a stronger behavioral NP effect in a study by Stadler and Hogan [53], did not show the increased response times in the present study. No significant differences were found for the contrast ((idDTTD – idC) vs. (idDT – idC)). However, fronto-striatal brain regions are significantly activated for DTTD trials, whereas they were only marginal significant for DT situations. Since a behavioral NP effect is lacking in DT and DTTD trials, further discussion of those observations would be only speculative. More studies on identity-based DT and DTTD tasks, which produce reliable behavioral NP effects, are necessary to understand hippocampal involvement in the framework of those studies.

Compared to control situations, DT/DTTD situations are processed differently on neural level. No differences were observed in behavioral measures. How these differences can be interpreted remains unclear. The assumption of the episodic retrieval account that retrieving processes cause prolonged response times is not fulfilled. The hippocampal activation cannot be clearly assigned to those retrieval processes described in the episodic retrieval account.

### Joint Activation Patterns

Statistical conjunction analysis did not give evidence for shared or common activation patterns for idDT and loDT. This means that idDT and loDT are operated differently in terms of brain function. Based on the strong generalizability of the behavioral NP effect, a commonly available ‘NP mechanism’ for both kinds of NP can be assumed. On the other hand there was no evidence that a double dissociation exists between idDT and loDT. IdDT minus loDT [(idDT – idC) – (loDT – loC)] resulted in marginal significant activation of the hippocampus. The reverse contrast [(loDT – loC) – (idDT – idC)] revealed marginal significant activation of the putamen. This indicates that the data of this study failed to demonstrate the existence of completely independent operating modes. It rather seems that loDT and idDT are processed by at least two independent mechanisms that are temporarily coupled. This is in accordance with observations made in comparative population studies, reporting a single dissociation.

The main issue of the current study was the investigation of brain activity during the performance of stimulus repetition tasks where distractor stimuli become target stimuli. This kind of stimulus repetition tasks has been intensively investigated in the framework of NP theories. Inhibition and episodic retrieval are part of current explanatory accounts of NP, trying to explain disfacilitation of responses on targets that have been presented as distractors before. Brain imaging has the potential to elucidate the role of inhibition and episodic retrieval for the processing of stimulus repetition tasks. Two variants of those tasks, identity-based and location-based tasks, have been discussed to be different with regard to the underlying processes despite the fact that both tasks show increased response times. Using a within-subjects design, both tasks were compared. Visual stimulus properties were held constant for both tasks. Results show that identity- and location-based tasks were in correlation with different patterns of brain activation. No shared activity was found for both tasks. However, location-based task performance was in correlation with fronto-striatal activation most likely indicating a predominance of inhibition processes. Identity-based task performance was associated with predominant hippocampal activation linking it to the concept of episodic retrieval. However, due to a lacking behavioral effect for only this kind of tasks, the interpretation of the sources of activation are limited. The DTTD variant of the tasks was thought to intensify switching from distractor to target. Results for DTTD showed basically the same activation pattern as the corresponding DT situation, differences only were expressed in slightly different statistical values. This study is the first one supporting explanatory accounts of NP using brain imaging for a direct comparison of the tasks under consideration. Multiple proposals for further research on the issue of this study have been made in the previous paragraphs.

## Methods

### Ethics Statement

The study was approved by the Institutional Review Board of the University of Giessen; procedures and measures were explained to the participants who provided informed consent before participating in the study.

### Participants

Twenty-eight subjects (15 male, 13 female; mean age: 25.36 years, *SD* = 4.33) participated in the study. All of them were students or had recently finished their studies. For participating in this fMRI study they gained 10 € or an equivalent (credits for participation in research).

### Experimental Paradigm

The experimental design was adopted from a study by Gibbons [3]. At the beginning of a session, subjects practiced the task outside the MR examination cabin. They were asked to conduct two tasks, an identity-based task and a location-based task.

**Figure 2:**
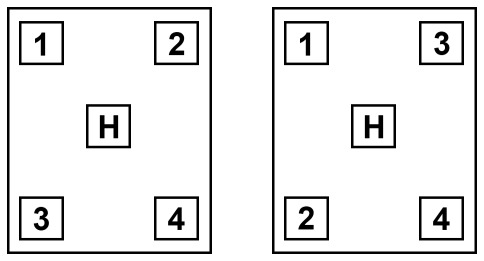
Exemplary illustration of arrangement of response buttons on the response pad. For the identity-based task, response buttons corresponded to the presented numbers on the screen. H = hold-over key.

During the identity-based task, the display was divided into four compartments, two of them containing a digit (range 1 to 4) – one in red color, the other one in blue. Subjects were asked to indicate the target digit, which could be recognized by the target color (red or blue, balanced over subjects). Responses were given by pressing the corresponding button on a four-button keypad with a holdover key in the middle. The buttons on the keypad were arranged according to the compartments on the display, each of them representing one digit. To avoid configuration effects, different configuration of the digits was used for the identity-based task. For one half of the subjects the upper left button corresponded to 1, the upper right to 2, the lower left to 3 and the lower right to 4. For the other half of the subjects the upper left button corresponded to 1, the lower left to 2, the upper right to 3 and the lower right to 4 (Figure 2). This was important for the identity-based task, in which subjects had to keep in mind the arrangement of the response buttons. In the location-based task, they had to press the response buttons according to the display, i.e. the upper right button to indicate that the target was presented in the upper right compartment of the display, etc.

Beside the control condition (‘C’), the DT condition and the DTTD condition, four other conditions sensu Christie and Klein [5] were implemented to avoid utilization of response strategies. Each trial began with a prime stimulus, which was presented until the subject had pressed one of the four buttons (limited to 1500 ms). The prime was followed by a fixation cross (presented for 200 ms). Subsequently, the corresponding probe stimulus was presented in the same manner as the prime stimulus. Finally, a fixation cross was displayed for 2000 – 4000 ms (jitter: 0 – 2000 ms).

**Figure 3:**
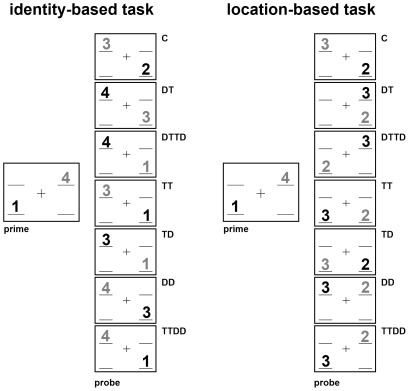
Exemplary illustration of the experimental conditions used in the experiment. In reality the numbers have been represented in red and blue, respectively.

Experimental conditions of both tasks were adopted from Christie and Klein [5], who supposed a fully balanced design in which the different targets are equally distributed over the identities (numbers 1 to 4)/locations (locations 1 to 4). Only the most important conditions were chosen for analysis. For the identity-based task, trials where the target stimulus in the probe had not been shown in the prime formed the control condition C. A trial was assigned to the DT condition, when the distracting stimulus in the prime was repeated as a target stimulus in the probe. In the DTTD condition, the distractor in the prime became the target stimulus in the probe, and the prime target became the probe distractor. Four additional conditions were implemented according to Christie and Klein [5]. These conditions did not enter the analysis. The TT condition consisted of trials in which prime targets were repeated as probe targets. In the TTDD trials, the prime target became the probe target, and the prime distractor became the probe distractor. In the TD trials, the prime target was shown as probe distractor; in the DD trials the prime distractor was shown as probe distractor (Figure 3). Each stimulus was equally often presented as target/distractor.

Both, the location-based task and the identity-based task rely on identical principles. The only difference was that subjects had to focus their attention either on stimulus location or on stimulus identity. In the location-based task the subjects were asked to indicate the location of the target stimulus (either the red or blue digit) by pressing the corresponding button (location) on the keypad. For this purpose, the arrangements of the buttons on the keypad and on the visual display were the identical. Analogously to the identity-based task, each arrangement of targets and distractors displayed in prime/probe matched one of seven conditions. Identification of targets and distractors depended solely on the location of the stimuli. The identity of the stimuli could be ignored.

An intermixture of the identity-based and location-based tasks was strictly avoided. The locations of the prime stimuli and the probe stimuli were never the same for identity-based tasks. Similarly, the prime stimuli had never the same identity as the probe stimuli in location-based tasks. This strict differentiation between identity- and location-based NP increases the predictability of the probe display. This might have an impact on results. For more discussion see Gibbons and Frings [56]. They found stronger inhibitory effects for identity-based DT situations when locations were unpredictable.

Each task (identity and location) consisted of 144 trials, of which 36 corresponded to condition C, and 18 to the experimental conditions (DT and DTTD), respectively. The additional conditions TT, TTDD, TD, and DD comprised 18 trials, respectively.

In order to control for sequence effects, the subjects were randomly assigned to two different trial sequences with equally distributed conditions over the time course of the task (restriction: not more than three identical subsequent conditions were allowed). All balancing factors were distributed over subjects as equally as possible.

The duration of each task amounted to about 11.5 minutes on average, depending on the individual subject’s speed of operation. Presentation software package (Neurobehavioral Systems, Albany, CA) was used to operate the task presentation. The stimuli were projected onto a backlight screen mounted near the MRI tube opening by an LCD projector. The subjects watched the screen by way of a head coil mounted mirror located approximately 20 cm above the subject’s eyes.

### fMRI Data Acquisition

Imaging data were acquired by a 1.5 T whole-body tomograph (General Electric; MR Signa NV/I). Structural image acquisition consisted of 172 T1-weighted sagittal images (MPRage, 0.8 mm slice thickness). To measure the blood oxygen level dependent (BOLD) contrast, a T2*-weighted single shot gradient echo EPI sequence (TR = 3 s, TE = 50 ms, flip angle = 90°, FOV = 240×240 mm^2^, 64×64 matrix) was used. One volume contained 30 slices with 5 mm slice thickness. The slices were acquired interleaved, in ascending order.

### Behavioral Data Analysis

Behavioral data were analyzed using the statistical software package Statistica 9 (StatSoft (Europe) GmbH, Hamburg). Response times exceeding 1300 ms or undershooting 300 ms were treated as outlier values and were therefore excluded from analysis of response times. For the analysis of response times with regard to the behavioral NP effect, only correctly answered trials were considered. Response time for each for the conditions idC, idDT, idDTTD, idTT, loC, loDT, loDTTD and loTT were calculated. Median response times (from stimulus onset until response) for each condition, was calculated for each subject. Median was used on subject level, because the usage of medians copes with the left-sided distribution of response times. On group level, the average of the median response latencies was built. For the analysis of main effects, a one-way ANOVA for repeated measures (including C, DT, DTTD, and TT) was conducted for the location-based task and the identity-based task separately. Subsequently, two-sided paired *t*-tests containing the four conditions were conducted for both tasks separately. We tested DT vs. C, DTTD vs. C, TT vs. C and DT vs. DTTD, respectively.

For correlation analysis with the fMRI data, the behavioral NP effect for the two tasks was calculated. For each subject, the median response time for the C trials was subtracted from the median response time for the DT trials.

### fMRI Data Analysis

FMRI data were analyzed using statistical parametric mapping methods with the SPM8 software package (Wellcome Department of Cognitive Neurology, London, UK) implemented in Matlab (Mathworks Inc., Sherborn, MA, USA). The first four volumes were discarded due to an incomplete steady state of magnetization. Preprocessing consisted of slice time correction (reference slice: 29), realignment (2nd degree b-spline interpolation to the mean image), and normalization to the standard space of the Montreal Neurological Institute (MNI) EPI template. Spatial smoothing was applied using an isotropic three-dimensional Gaussian filter with a full width at half maximum of 8 mm to allow for corrected statistical inference.

The evoked BOLD responses were modeled for the 14 conditions (for the identity-based task: C, DT, DTTD, as well as TT, TD, DD, and TTDD; for the location-based task: C, DT, DTTD, as well as TT, TD, DD, and TTDD). Regressors representing the experimental conditions were built using the exact duration of each single event, which was defined as time from onset of the prime stimulus to the subject’s response on the probe stimulus. Due to increased response times for the identity-based task, durations for idDT were in average 400 ms longer in comparison to those for loDT. We are able to demonstrate that this difference had no impact on BOLD sampling. In the appendix, exemplary averaged hemodynamic responses for selected ROI are displayed (Figure S1). Finally, regressors were convolved with a canonical hemodynamic response function to serve the hemodynamic signal characteristics. The computation of time or dispersion derivations was not indicated. As commonly recommended, the six movement parameters derived from the realignment pre-processing step were added to the model to control for residual movement related variance. A high pass filter was set to time constant = 128 s to reduce slowly changing artifacts of technical or biological origin. The existence of serial correlation, which violates pre-conditions of the ALM, was controlled using autoregressive AR1-estimations.

Whole-brain analyses revealed no significant results at *p_corr_*<.05, *k* = 0 (FWE-corrected). Region of interest (ROI) analyses included only a priori chosen brain regions. Selection was based on the relevant literature reported in the introduction paragraph with emphasis on retrieval and inhibition processing, particularly for DT and DTTD trials: pars opercularis and pars triangularis, ACC, hippocampus, striatum (pallidum, putamen, NC). ROI analyses were conducted separately for each hemisphere. The corresponding ROI masks were generated using the AAL-atlas, which can be found within the WFU PickAtlas, an automated software toolbox for generating ROI masks based on the Talairach Daemon database [57]–[60]. The PickAtlas automatically considers the SPM small volume correction, giving *p*-values corrected for multiple comparisons. All reported ROI results were tested at *p_corr_*<.05 and adjusted according to the gaussian random field theory to control for the family-wise error (FWE). For ROI analyses all *T*-values and family-wise error corrected *p*-values are listed. Detailed information about all the methodical and statistical issues can be found at http://www.fil.ion.ucl.ac.uk/spm/.

Just as for the response times, we included C, DT and DTTD in the analysis of the neural data. The hypothesis that idDT and loDT are processed by identical networks was tested using SPM8 conjunction analysis (idDT – idC) ∩ (loDT – loC). For the direct comparison of idDT and loDT both the contrast (idDT – idC) – (loDT – loC) and the inversed contrast (loDT – loC) – (idDT – idC) were computed. All contrasts were referred to C to cope with possibly different response modes for loDT and idDT. For idDT, subjects had to remember the position of the button for each specific number stimulus (cf. Figure 2). For loDT the arrangement of the default locations on the display and of the response buttons was identical. The contrast (DT – C) was used to study neural activation patterns involved in DT trials separately for the identity-based and the location-based task. Adjacently, we tested for direct positive correlations between the BOLD and the behavioral NP effect (see section ‘Behavioral Data Analysis’ for the calculation) in those ROI, which we found marginal significantly (*p_corr_*<.1) or significantly activated in the contrasts (idDT – idC) and (loDT – loC). As mentioned previously, one of the two most discussed theories explains the NP effect being based on retrieval. Repetition of stimuli induces retrieval; therefore an additional analysis was performed including the TT condition where the identical target stimulus is presented consecutively. This analysis helps to decide if hippocampal activation is initiated by purely stimulus repetition or is dedicated to NP. The contrasts (loDTTD – loC) and (idDTTD – idC) were investigated to analyze the condition DTTD. The difference between DTTD and DT was assessed by the contrasts ((loDTTD – loC) – (loDT – loC)) and ((idDTTD – idC) – (idDT – idC)).

## Supporting Information

Figure S1 **Event-averaged BOLD signal for the DT conditions.** Data for the identity-based priming task are illustrated in continuous blue lines, data for the location-based priming task in dotted red lines.
(TIF)
