# Supplementary figures and images for: Neuroimaging Evidence for Processes Underlying Repetition of Ignored Stimuli
Source: PLoS One. 2012 May 1;7(5):e36089. doi: 10.1371/journal.pone.0036089 (PMC3341391; doi:10.1371/journal.pone.0036089)

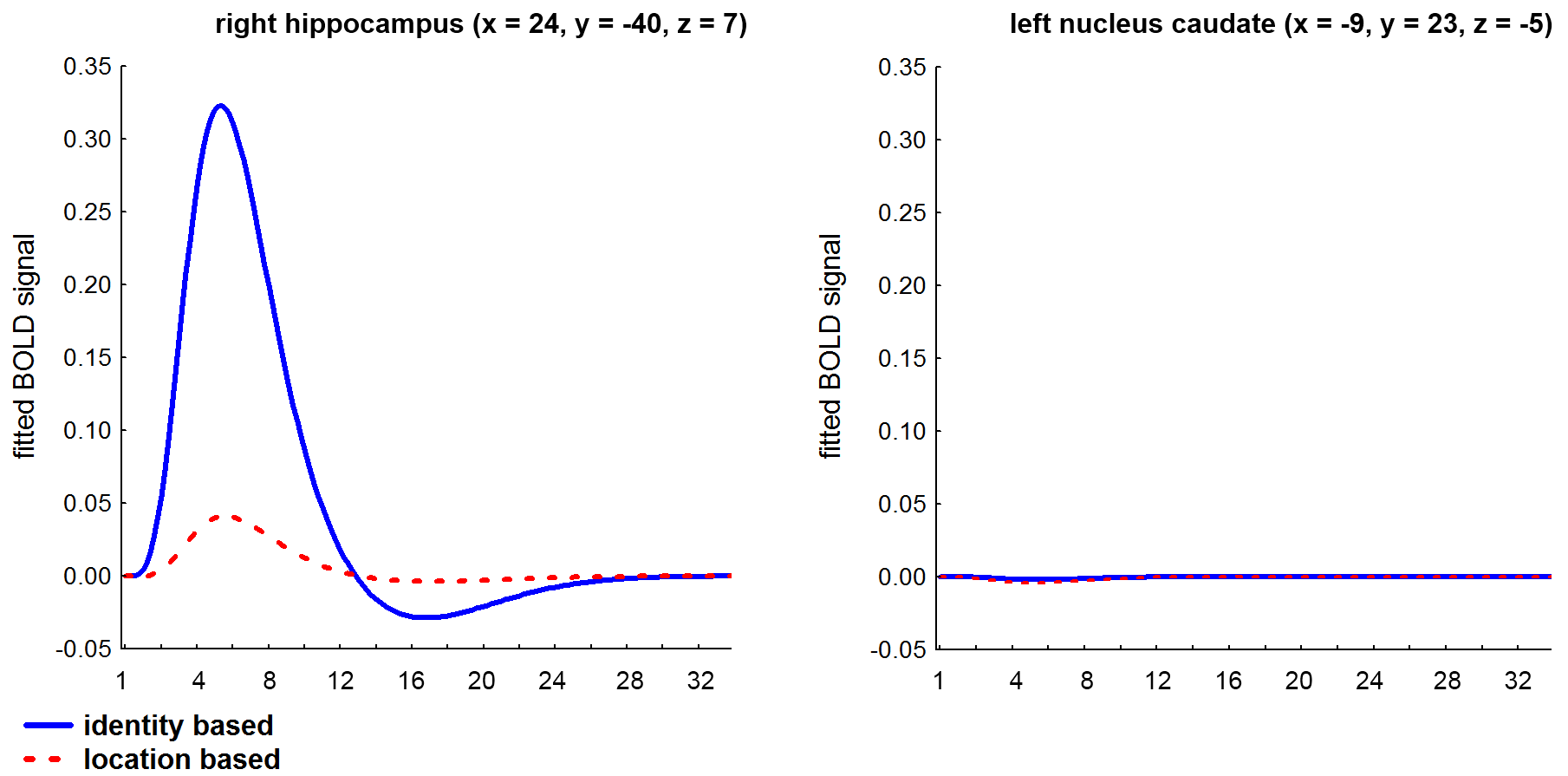

Supplement: Figure S1 — Event-averaged BOLD signal for the DT conditions. Data for the identity-based priming task are illustrated in continuous blue lines, data for the location-based priming task in dotted red lines. (TIF) [file pone.0036089.s001.tif]
